# A novel gene organization of the rock sparrow *Petronia petronia* (Aves: Passeriformes) revealed by complete mitochondrial genome

**DOI:** 10.1080/23802359.2017.1407709

**Published:** 2017-11-25

**Authors:** Ruirui Shi, Kai Chen, Shaobin Li

**Affiliations:** a Institute of Biomedicine, College of Life Science, Yangtze University, Jingzhou, China;; b Ecological Security and Protection Key Laboratory of Sichuan Province, Mianyang Normal University, Mianyang, China

**Keywords:** Mitochondrion, novel gene, passerine, *Petronia petronia*, Tibet plateau

## Abstract

The whole sequence of the Rock Sparrow (*Petronia petronia*) mitochondrial genome was determined using La-PCR and conserved primer walking approaches. The entire mitogenome was 17,426 bp in length and harboured 13 protein-coding genes, 2 rRNA genes, 23 tRNA genes, and 1 noncoding control region. The mitogenome of this species resembled other avian species in gene arrangement and composition, except that *tRNA-Glu* had two copies and the control region contained two parts. One part of the control region is between *tRNA-Glu* and *tRNA-Phe* and a second between the two *tRNA-Glu* copies. The overall nucleotide composition are A (30.7%), T (23.8%), G (14.4%), and C (31.1%) with 54.5% A + T content. These mitochondrial data are potentially important for the study of molecular evolution and conservation genetics.

The Rock Sparrow (*Petronia petronia*) classified in the family Passeridae is an old world sparrow (Zheng 2005; Gill and Donsker 2017; Summers-Smith 2017). The birds generally occurred in bare treeless habitats from Asia to Europe and placed their nest in cavity (Li and Lu 2012; Gill and Donsker 2017). Their altitudinal distributions ranged from sea level to more than 4000 m altitude (Li and Lu 2012; Summers-Smith 2017). The biology and ecology of this species has been well studied in both Europe and Asia (Tavecchia et al. 2002; Griggio et al. 2011; Li and Lu 2012), but its complete mitochondrial genome has not been reported. In this study, we sequenced the complete mitochondrial genome of *P. petronia* and the newly sequenced mitochondrial genome sequence will provide basic data for further molecular phylogenetic studies.

Blood sample of the Rock Sparrow was collected by puncturing the brachial vein from a male individual (id Rs_06) caught by mist net, on 19 July 2016 at Tianjun County (37°18′ N, 99° 01′ E; 3430 m a.s.l.), northeastern of Tibetan Plateau. Genomic DNA was extracted from blood samples according to the protocol of TIANamp Genomic DNA kits (Tiangen, Beijing). The blood sample is stored in the Room 420 of College of Life Science at Yangtze University. The complete sequence of the Rock Sparrow mitochondrial genome was determined using long-range PCR and conserved primer walking approaches.

The entire mitochondrial genome of the Rock Sparrow was 17,426 bp in length (GenBank accession number MF071218) and contained 13 protein-coding genes, 2 rRNA genes, 23 tRNA genes, and a non-coding control region. The overall nucleotide composition are A (30.7%), T (23.8%), G (14.4%), and C (31.1%), with a total A and T content of 54.1%. The mitogenome of *P. petronia* resembled other avian species in gene arrangement and base composition, except that *tRNA-Glu* had two copies and the control region contained two parts. The control region was similar to other avian species but the whole sequence of *tRNA-Glu* was duplicated in a 530-bp fragment of the control region. Therefore, unlike other avian species, *P. petronia* had a second part of the control region between the two *tRNA-Glu* copies. This case is a rarity, but it is by no means unique in vertebrate (Li et al. 2015).

Among the 38 genes, 28 were encoded on heavy strand (similar as other avian species) and the remaining genes on the light strand. All protein-coding genes of the *P. petronia* mitochondrial genome started with ATG codon, except for *COI* and *ND2* which were started with GTG and ATA, respectively. For terminate codon usage, most of the genes terminate with codons TAA or TAG, and *ND5* terminated with AGA, *ND1* and *COXI* with AGG, and the *COXIII* and *ND4* genes had an incomplete termination codon T—.

Phylogenetic analyses were conducted with mitochondrial genomic data of 26 avian species (including the Rock Sparrow) from the GenBank database. The topology of the tree inferred using Neighbour-Joining methods in the program MEGA7 (Kumar et al. 2016). Execution model was statistically well supported by high bootstrap values at most nodes (Figure 1). The phylogenetic analysis support the bird species classification that the Rock Sparrow was closely related to *Montifringilla* species than *Passer* species (Clements et al. 2016; Gill and Donsker 2017). Blast results in GenBank were consistent with our phylogenetic analysis. This complete chloroplast genome of the Rock Sparrow could be fundamental to further molecular phylogenetic studies on avian species.

**Figure 1.**
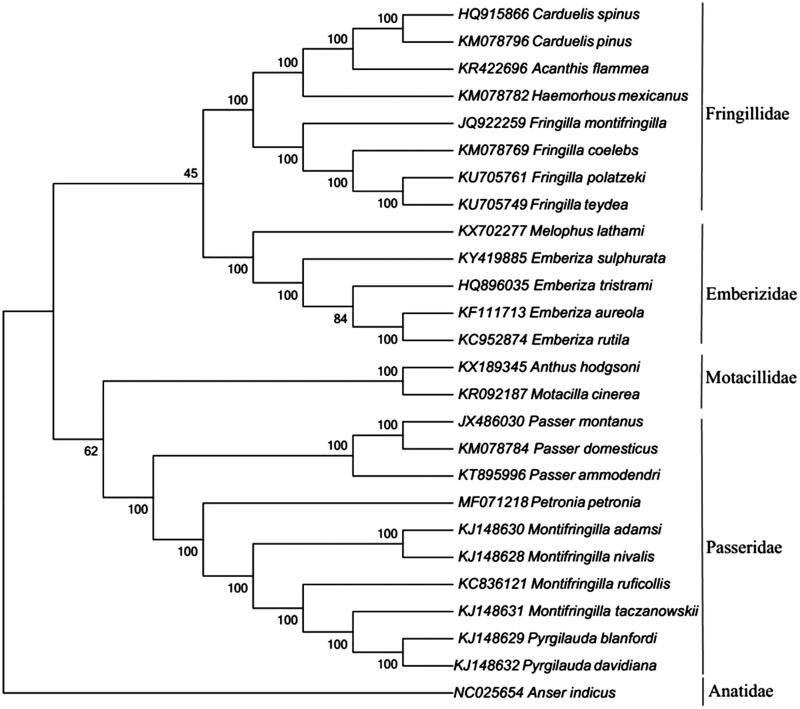
Molecular phylogenetic tree of 26 passerine bird species constructed with Neighbour-Joining method based on their complete mitochondrial genomes with the accession number in parentheses. The bootstrap values are based on 1000 resamplings. The *number at each node* is the bootstrap probability. The *number* before the species name is the GenBank accession number.
